# Optical-referenceless optical frequency counter with twelve-digit absolute accuracy

**DOI:** 10.1038/s41598-023-35674-8

**Published:** 2023-05-30

**Authors:** Atsushi Ishizawa, Tadashi Nishikawa, Kenichi Hitachi, Tomoya Akatsuka, Katsuya Oguri

**Affiliations:** 1 NTT Basic Research Laboratories, Nippon Telegraph and Telephone Corporation, 3-1 Morinosato Wakamiya, Atsugi, Kanagawa 243-0198 Japan; 2 Department of Electronic Engineering, Tokyo Denki University, 5 Senjyu-Asahi-cho, Adachi-ku, Tokyo, 120-8551 Japan; 3 Present Address: College of Industrial Technology, Nihon University, 1-2-1 Izumi-cho, Narashino, Chiba 275-8575 Japan

**Keywords:** Frequency combs, Supercontinuum generation

## Abstract

A simpler and more accurate measurement of absolute optical frequencies (AOFs) is very important for optical communications and navigation systems. To date, an optical reference has been needed for measuring AOFs with twelve-digit accuracy because of the difficulty in measuring them directly. Here, we focus on an electro-optics-modulation comb that can bridge the vast frequency gap between photonics and electronics. We demonstrate an unprecedented method that can directly measure AOFs to an accuracy of twelve digits with an RF frequency counter by simply delivering a frequency-unknown laser into an optical phase modulator. This could open up a new horizon for optical-referenceless optical frequency metrology. Our method can also simultaneously achieve a 100-fold phase-noise reduction in a conventional signal generator. This corresponds to an increase in the transmission speed of wireless communications of by about seven times.

## Introduction

The growing demand for low-phase-noise microwave generation at unprecedented levels in coherent radar systems^1, 2^, phase/clock synchronization^3–5^, and high-speed analog-to-digital conversion^1, 6, 7^ has been creating challenges in microwave-photonics technologies^8^. In radar systems, a 10-GHz microwave with an ultra-low phase noise of − 170 dBc/Hz at 10-kHz offset frequency is required for tracking small objects such as drones. In phase/clock synchronization, microwave signals with low phase noise have become increasingly important for e-commerce, such as high-frequency trading and trusted time stamping^5^, electrical power systems such as smart grids^9^, and distributed processing in data centers. For more accurate phase/clock synchronization^10, 11^, optical clocks, such as optical lattice and ion clocks, have been discussed in ITU-T as the future master clocks^12^. SDH (Synchronous Digital Hierarchy) and SONET (Synchronous Optical Network) are standard protocols for digital communications networks that use optical fibre. The basic frame size of SDH/SONET is defined as 125 µs per frame^13^. The frequency accuracy of the current cesium master clocks is 10^–11^. If two communications devices synchronized with different cesium master clocks perform data read and write, the current slip interval for reading digital signals occurs every 72 days. In contrast, the optical lattice clock (frequency accuracy: 10^–18^) can make the slip interval two million years, so it will be a maintenance-free master clock. Since telecommunications systems work at frequencies from gigahertz to kilohertz, the optical clock frequency (sub-petahertz) of a master clock will have to be precisely down-converted. Some microwave generation methods based on photonic technologies, such as whispering-gallery-mode parametric oscillators^14^, optical frequency division^15–17^, optoelectric oscillators^18^, on-chip Brillouin oscillators^19^, and optical reference cavities^20^, have been reported. A recent study showed ultralow-noise microwaves can be generated with a frequency comb based on an ultralow-noise mode-locked fibre laser^21^. This method achieves excellent low-noise microwave generation at 12 GHz, but it would be difficult to provide end users with a complex apparatus comprising many sets of large, low-noise fibre-laser-based frequency combs.

In the field of optical frequency metrology, it had been impossible to directly measure the AOF using an RF frequency counter because the optical frequency is about tens of thousands of times higher than the microwave frequency. Prior to 1999, AOF counters used an optical frequency chain^22–24^, which measured high frequencies by sequentially multiplying and mixing low frequencies. The measurement required a lot of stable lasers, microwave oscillators, and wavelength conversion elements in addition to control circuits and measurement tools. In 1999, the optical frequency comb (OFC)^25–28^ appeared, which dramatically shifted attention away from the complex optical frequency chain. The frequency of the *N*th comb tooth, *f*_N_, can be expressed as ${f}_{ceo}+N\times {f}_{rep}$, where *N*, *f*_rep_, and *f*_ceo_ are the comb mode number, repetition frequency, and carrier-envelope-offset (CEO) frequency, respectively. To measure the AOFs of a frequency-unknown laser using an OFC, beat frequency ${f}_{b}$ between the *N*th comb tooth and the frequency-unknown laser is measured. Thus, $f$, is described as ${f}_{ceo}+N\times {f}_{rep}\pm {f}_{b}$. In practice, the comb mode number *N* can be determined by measuring the comb mode number closest to the unknown laser source. This can be done either by using a wavelength meter with sufficient precision and accuracy to measure the OFC within *f*_rep_/2, or by measuring the *f*_rep_ and *f*_b_ and counting the change in comb mode number while varying *f*_rep_ by a large amount, typically on the order of MHz. The former method requires a highly accurate wavelength meter and an optical frequency comb as an optical reference source, while the latter method only requires an optical frequency comb as the optical reference source. However, the latter method can be complicated as it requires accurate counting of the change in comb mode number while varying *f*_rep_ by a large amount.

Here, we demonstrate a simpler optical-referenceless optical frequency counter. With our method, an AOF can be directly measured to twelve-digit accuracy with an RF frequency counter by simply delivering a frequency-unknown laser into a phase modulator without relying on any optical reference source. Furthermore, with the use of a highly frequency-stable light source in our method, a 100-fold phase-noise reduction in conventional widely used signal generators (SGs) can be simultaneously achieved by magnifying an SG’s phase noise in the optical frequency region with an electro-optics-modulation (EOM) comb and feeding it back to the SG. From the Shannon-Hartley theorem, this means that the transmission speed of wireless communications can be increased by about seven times.

## Phase-noise reduction with CEO signal as a “phase-noise booster”

The phase noise of an EOM comb^29–31^ mainly originates from that of the SG used to drive phase/intensity modulators. The mode number in an EOM comb is defined as the number of comb modes from the center frequency (mode number 0) of a seed light source. The phase noise of an SG as well as the linewidth of the EOM comb modes is magnified as the comb mode number increases^32^.

**Figure 1:**
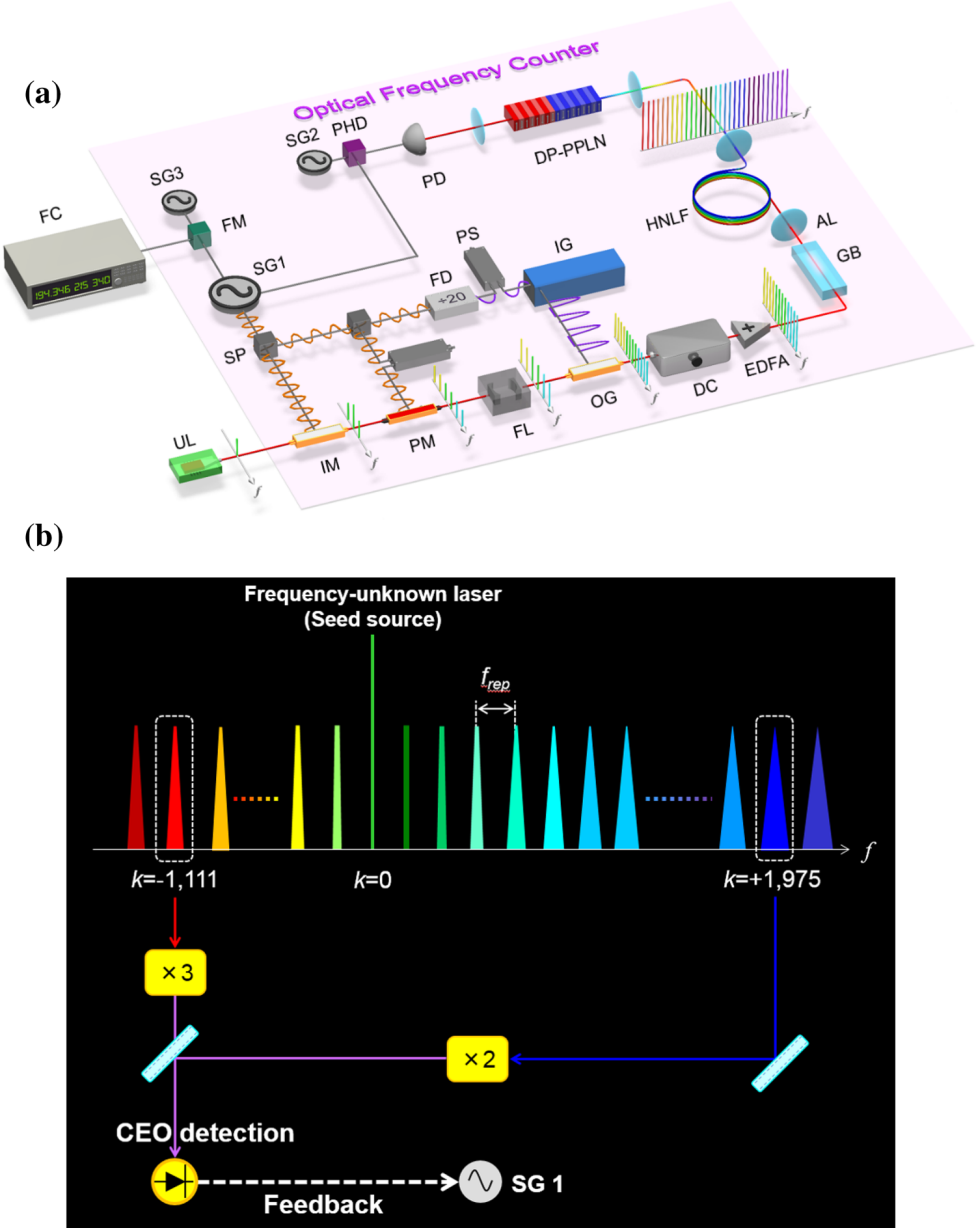
(**a**) Experimental setup for the optical-referenceless AOF counter. SG: Signal generator. All three SGs and FC are referenced to the common GPS-disciplined RF source. UL: Frequency-unknown laser. IM: Intensity modulator. PM: Phase modulator. SP: Splitter. PS: Phase shifter. FD: Frequency divider. FL: Filter cavity. IG: Impulse generator. OG: Optical gate. DC: Dispersion controller. EDFA: Er-doped fibre amplifier. GB: Glass block. AL: Aspherical lens. HNLF: Highly nonlinear fibre. DP-PPLN: Dual-pitch-periodically poled lithium niobate ridge waveguide. PD: Photodetector. PHD: Phase detector. FM: Frequency mixer. FC: Frequency counter. (**b**) Our concept of low-phase-noise microwave generation with CEO signal.

Therefore, the CEO signal, which is a beat note between the high-order comb modes, includes the boosted SG’s phase-noise information. Figure 1a shows our experimental setup for decreasing the phase noise of a commercially available SG (SG 1) with a PLL feedback circuit (see Methods for details). SG 1 is synchronized with a reference signal from a global-positioning-system-(GPS)-disciplined BVA oven-controlled crystal oscillator (OCXO) (frequency instability: < 3 × 10^–13^ @ 1 s). A 25-GHz optical pulse train is generated by phase-modulating an ultra-stable laser (frequency instability: 1 × 10^–15^ @ 1 s) with a center wavelength of 1,542 nm and a linewidth of 1 Hz. After the repetition rate is decreased to 1.25 GHz with an optical gate, the laser light is amplified with an EDFA up to 1 W. Supercontinuum spectra with more than a 2/3-octave bandwidth can be successfully generated by using a 40-cm-long highly nonlinear fibre. Figure 1b shows our concept of low-phase-noise microwave generation without relying on any optical reference. We detect the CEO beat signal by injecting the output from a collinear 2*f*-to-3*f* self-referencing interferometer (SRI) into a photodetector. The EOM comb mode number is defined as the number of comb modes from the ultra-stable laser (mode number 0). The CEO signal includes the information for boosting the phase noise of SG 1 by up to (2 × 1,975) + (3 × 1,111) = 7,283 times because it is the beat note between the + 1,975th and -1,111st comb modes with a 2*f*-to-3*f* SRI. The CEO signal has large phase fluctuation because it is generated from the beat signal between high-order comb modes. After dividing the CEO frequency by 32, we detect the phase difference between the CEO signal and an external reference RF signal from SG 2, which is synchronized with the reference signal from the GPS-disciplined BVA OCXO. The low-frequency component is then selected with a lowpass filter. The YIG-oscillator-based VCO inside SG 1 adjusts the voltage so that the phase difference becomes zero (see “Supplementary Information” for details). Finally, the phase noise of SG 1 can be greatly reduced. In previous work^33^, reducing the SG phase noise required a mode-locked laser as the optical reference. The present method with the CEO signal can greatly reduce the SG phase noise without relying on any optical reference.

**Figure 2:**
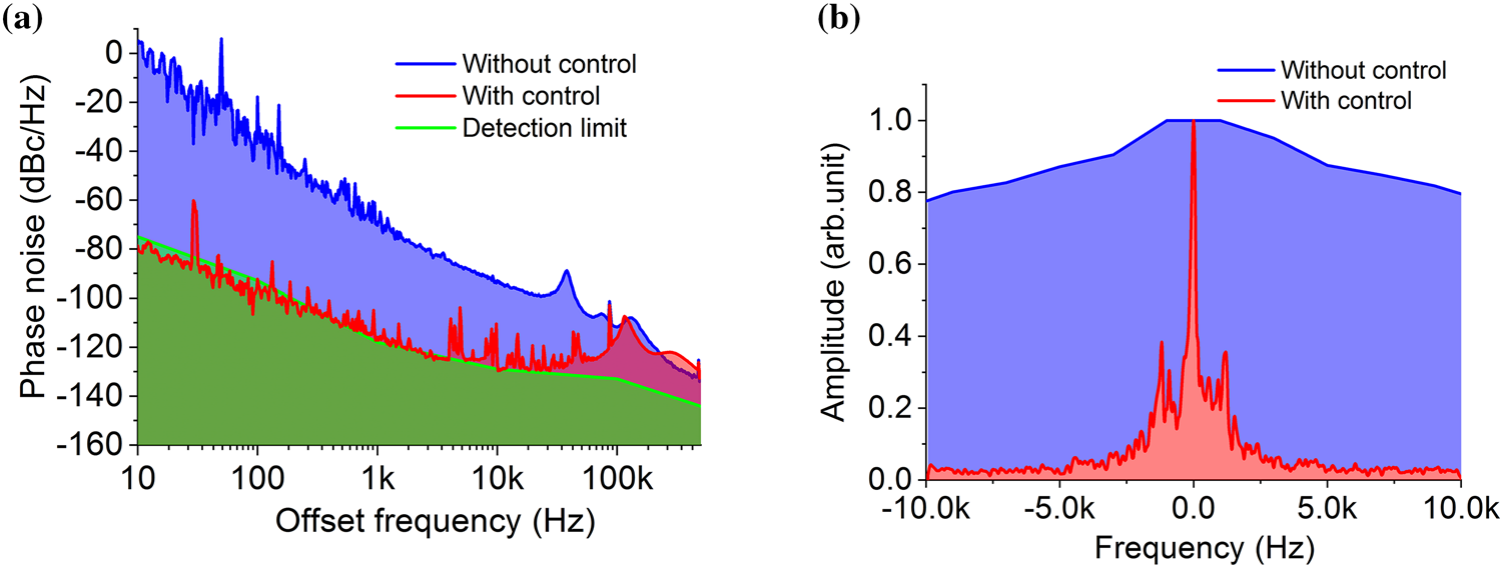
(**a**) Measured phase noise of SG 1 with and without feedback using the CEO signal. (**b**) Beat note between the 811th mode of the EOM comb and an ultra-stable laser at 1,397 nm with and without control using the CEO signal.

We experimentally demonstrated, for the first time, that phase noise *φ* (t) of SG 1 at around 25 GHz can be lowered by PLL feedback with the CEO signal (see Fig. 2a). Since the feedback loop bandwidth is set at 300 kHz, *φ* (t) can be reduced at an offset frequency of less than 300 kHz. Under our experimental conditions, *φ* (t) can be greatly reduced to the limit of the phase-noise detection with a low noise floor and cross-correlation measurement (E5052B + E5053A, Keysight Technology), and the lowest *φ* (t) value is -130 dBc/Hz at an offset frequency of 10 kHz. The phase noise we achieved with SG 1 is far lower than the lowest phase noise reported for commercially available SGs with the reference signal from the GPS-disciplined BVA OCXO. In Fig. 2b, we show the beat signal between the EOM comb and an ultra-stable laser with a center wavelength of 1,397 nm, a linewidth of 1 Hz, and frequency instability of 1 × 10^–15^ @ 1 s. One can see that the EOM comb linewidth at the 811st comb mode number is narrowed to about 300 Hz by reducing SG 1’s phase noise with our method. These results indicate that our method can achieve both low-noise microwave generation and a narrow linewidth EOM comb with 25-GHz mode spacing.

## AOF measurement without an optical reference

**Figure 3:**
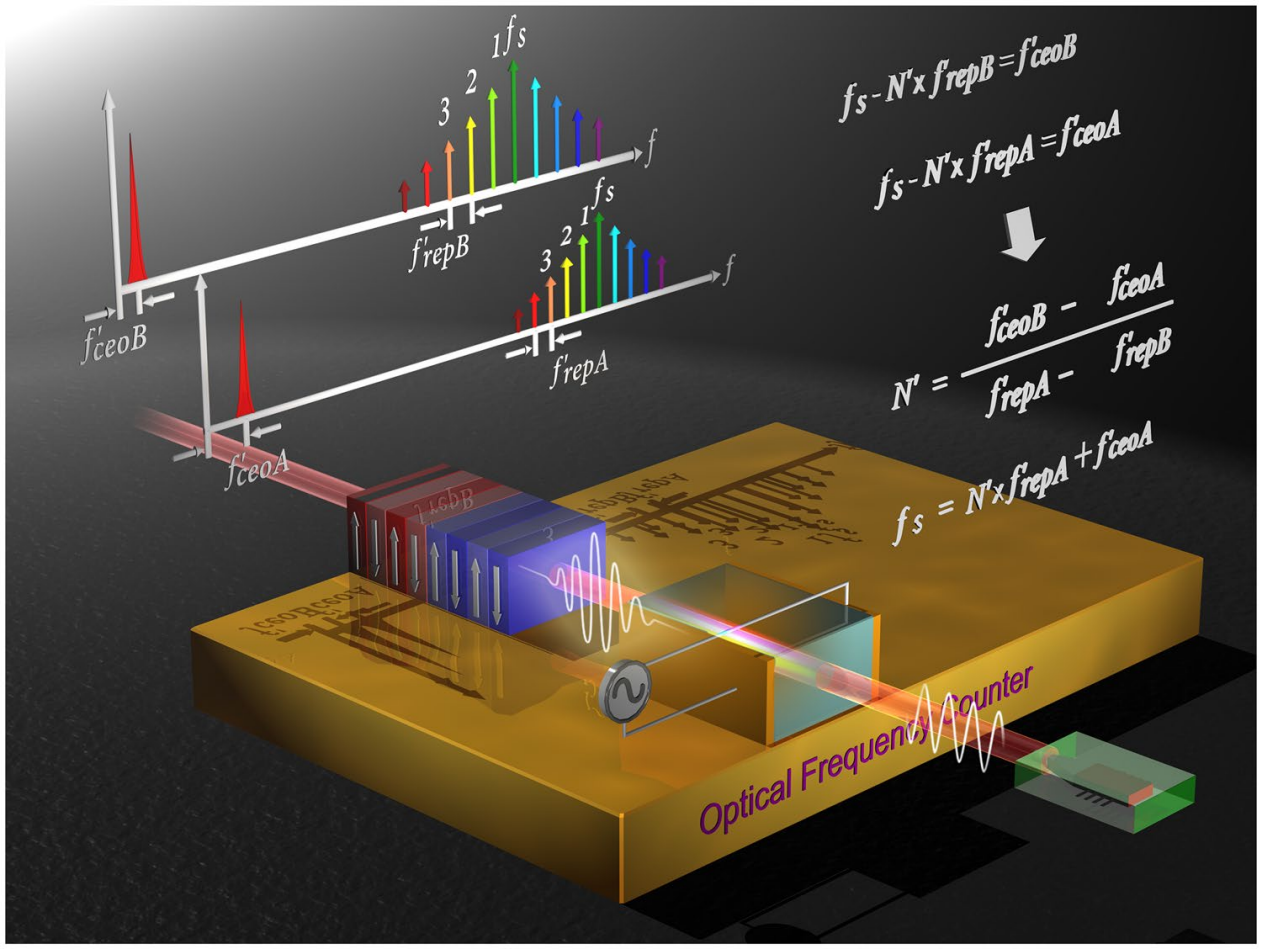
**Concept of optical-referenceless optical frequency counter.** In this optical-referenceless method, the AOF of a frequency-unknown laser with twelve-digit accuracy can be directly measured by using an RF frequency counter.

OFCs have revolutionized the field of optical frequency metrology. Here, we demonstrated AOF measurement using an EOM comb seeded by a frequency-unknown laser without relying on an optical reference source. Figure 3 shows our concept of the optical-referenceless optical frequency counter. As mentioned in the previous section, ${f}_{rep}$ can be determined by the output frequency of SG 1 and ${f}_{ceo}$ can be measured with an SRI. Furthermore, the EOM comb can easily generate an OFC with variable mode spacings of more than 10 GHz. Therefore, the EOM comb becomes a simple and easy-to-use tool for determining mode number $N$ without using a high-precision wavelength meter or an optical reference source. The details of the method are as follows.

In this experiment (see setup in Fig. 1a), we measured the AOF ${f}_{s}$ of the ultra-stable laser with a linewidth of 1 Hz, which was used as the seed laser source for the EOM comb. As shown in the previous section, the EOM comb is generated, and then the CEO signal ${f^{\prime}}_{ceo}$ after the optical gate (the prime symbol indicates the frequency after the optical gate) can be measured. The output frequency of SG 1 can be stabilized by detecting the phase difference between the CEO signal ${f^{\prime}}_{ceo}$ and SG 2’s signal and feeding it back to SG 1. To measure repetition frequency ${f^{\prime}}_{rep}$ after the optical gate with a large number of digits with a frequency counter, we measured the difference frequency between the frequency-divided signal (see purple sine wave in Fig. 1a) of SG 1, which drives the optical gate, and output frequency ${f}_{ex}$ of SG 3 (set at $1.249 999 \mathrm{GHz}$). SG 1, SG 2, and SG 3 were synchronized with the reference signal from the GPS-disciplined BVA OCXO.

We controlled SG 1 so that the phase difference between SG 2’s signal and the measured CEO signal became zero by using the feedback circuit. When we set the output frequency of SG 2 as ${f^{\prime}}_{ceoA}$, the measured difference frequency between the frequency-divided signal of SG 1 and ${f}_{ex}$ is ${\Delta f^{\prime}}_{repA}$. Similarly, when we set the output frequency of SG 2 as ${f^{\prime}}_{ceoB}$, the measured difference frequency is ${\Delta f^{\prime}}_{repB}$. If the drift of optical frequency of the seed laser source, ${f}_{s}$, is sufficiently small during the measurement,

1 $${f}_{s}={{f}^{\prime}}_{ceoA}+{N}^{\prime}\times {{f}^{\prime}}_{repA}={{f}^{\prime}}_{ceoA}+N^{\prime}\times {({f}_{ex}+\Delta {f}^{\prime}}_{repA})$$

2 $${f}_{s}={{f}^{\prime}}_{ceoB}+{N}^{\prime}\times {{f}^{\prime}}_{repB}={{f}^{\prime}}_{ceoB}+N^{\prime}\times {({f}_{ex}+\Delta {f}^{\prime}}_{repB})$$

By using both (1) and (2), mode number $N^{\prime}$ after the optical gate can be obtained as follows (see Fig. 3):

3 $${N}^{\prime}=\frac{{f^{\prime}}_{ceoB}-{f^{\prime}}_{ceoA}}{{f^{\prime}}_{repA}-{f^{\prime}}_{repB}}=\frac{{f^{\prime}}_{ceoB}-{f^{\prime}}_{ceoA}}{\Delta {f}_{repA}^{\prime}-\Delta {f}_{repB}^{\prime}}.$$

In our experiment, when we locked ${f^{\prime}}_{ceoA}$ to + 130 MHz, $\Delta {f}_{repA}^{\prime}$ was measured as $970.029 969 118 \mathrm{Hz}$, and when we locked ${f^{\prime}}_{ceoB}$ to -120 MHz, $\Delta {f}_{repB}^{\prime}$ was measured as $2577.984 362 83 \mathrm{Hz}$. Therefore, we could determine mode number ${N}^{\prime}$ after the optical gate as 155 477. Next, we calculated the optical frequency of the seed laser source (= ultra-stable laser), ${f}_{sA}$ and ${f}_{sB}$, using two sets of (${N}^{\prime}$, ${f^{\prime}}_{ceoA}$,and $\Delta {f}_{repA}^{\prime}$) and (${N}^{\prime}, {f^{\prime}}_{ceoB}$,and $\Delta {f}_{repB}^{\prime}$). We confirmed that ${\Delta {f}^{\prime}}_{repA}$ and ${\Delta {f}^{\prime}}_{repB}$ have positive signs for the output frequency ${f}_{ex}(=1.249 999 \mathrm{GHz})$ of external RF reference SG 3. In addition, we also found that our CEO signal for measurement is not ${{f}^{\prime}}_{rep}-{{f}^{\prime}}_{ceo}$ but ${{f}^{\prime}}_{ceo}$. Therefore, ${f}_{sA}$ and ${f}_{sB}$ are calculated as

$$\begin{aligned} f_{sA} & = 130 \,{\text{MHz}} + 155477 \times (1.249999\,{\text{GHz}} + 970.029 969 118\,{\text{Hz}}) \\ & = 194. 346 375 340 350\,{\text{THz}} \\ \end{aligned}$$

$$\begin{aligned} f_{sB} & = - 120\,{\text{MHz}} + 155477 \times (1.249999\,{\text{GHz}} + 2577.984 362 83\,{\text{Hz}}) \\ & = 194. 346 375 340 275\,{\text{THz}}. \\ \end{aligned}$$

In this way, we can determine the AOF of the seed laser, ${f}_{s},$ as $194.346 375 340(1) \mathrm{THz}$ with twelve-digit accuracy.

If large optical frequency fluctuation of the frequency-unknown laser occurs during the measurement, the mode number cannot be obtained with this method. The use of the method is limited to the case when frequency fluctuations are less than ${{f}^{\prime}}_{rep}/2$ after the optical gate that is as long as the comb mode number stays the same during the measurement time. In such a case, we need to use a stable laser with small optical frequency fluctuation as the seed laser of the EOM comb. After the AOF of the stable laser ${f}_{s}$ is determined with the above method, we observe beat frequency ${f}_{b}$ between the frequency-unknown laser and EOM comb with wide mode spacing (25 GHz) before the optical gate. As a result, the AOF of the frequency-unknown laser, $f$, is described as.

$$f = f_{s} + M \times f_{rep} \pm f_{b} .$$

The sign before ${f}_{b}$ can be easily determined by slightly shifting seed laser frequency ${f}_{s}$. Mode number $M$ can be easily determined by using a conventional wavelength meter with wavelength accuracy of less than 25 GHz.

A recently reported technique uses a Si_3_N_4_ wire waveguide to detect the CEO signal with ultra-low laser pulse energy^34–36^. With this technique, the frequency-divided ratio of the optical gate can be decreased and a CEO-locked EOM comb with wider mode spacing can be achieved. In this case, it will be possible to determine mode number $N^{\prime}$ will become much simply by using a low-accuracy wavelength meter, and then the AOF of the frequency-unknown laser with twelve-digit accuracy could be calculated easily.

## Super simple optical synthesizer based on a free-running continuous-wave laser diode

**Figure 4:**
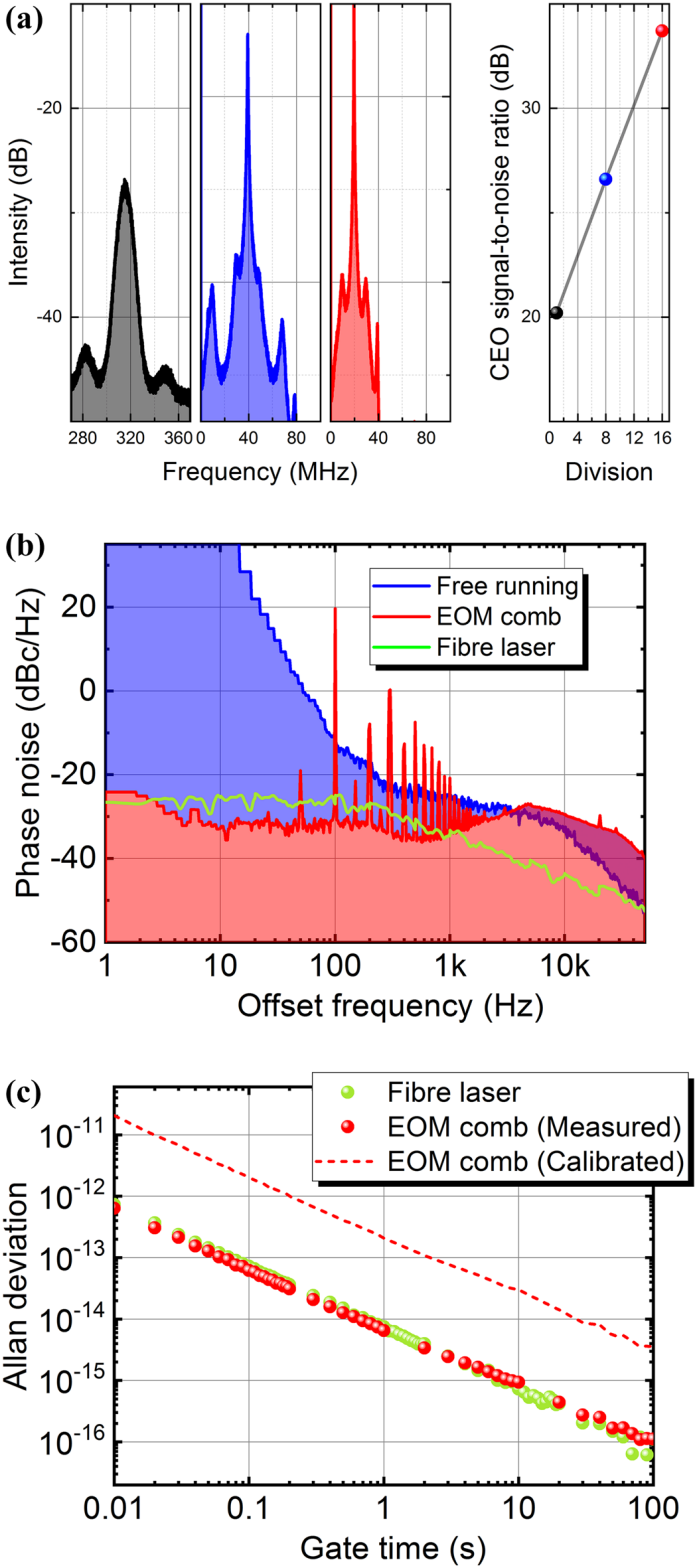
(**a**) The left graph shows the CEO spectra measured with an RF spectral analyzer, which are frequency-divided by 1 (black), 8 (blue), and 16 (red), respectively. The right graph shows the frequency-dividing dependency of the SNR of the CEO signal. (**b**) Measured CEO phase noise with (red) and without (blue) the CEO locking using the EOM comb. We compared the phase noise with the CEO locking using a commercially available mode-locked fibre laser (green). (**c**) Measured Allan deviation of CEO signal with the CEO locking of the commercially available mode-locked fibre laser (green). The red circle and dotted line show the measured Allan deviation for the CEO frequency divided by 32 and the calibrated Allan deviation for the CEO frequency, which is frequency-divided by 1 [see a black line in (**a**)] with the EOM comb. The Allan deviation of the CEO signal is shown on the vertical axis, divided by the center optical frequency (mode number 0). The gate time, which is the length of time that the frequency counter counts a signal, is shown on the horizontal axis.

CEO-locked frequency combs with high frequency stability and high coherency are very attractive for future photonic network systems and dual-comb spectroscopy^37, 38^. Recent research and development of optical fibre transmission technologies has been shifting from simple intensity modulation to multi-level intensity and phase modulation to achieve high spectral utilization efficiency^39^. This has led to the need for an optical light source with high frequency stability and coherence. The optical carrier light sources are assigned to a frequency grid, which has been standardized in ITU-T as integer multiples of 12.5-, 25-, 50-, and 100-GHz at the anchor frequency of 193.1 THz^40^. The light is transmitted through optical fibre by using dense wavelength-division multiplexing (DWDM) technology. Thus, an OFC which indicates the ITU-T frequency grid will be needed in future photonic network systems. However, it is difficult to achieve a CEO-locked frequency comb at telecommunications wavelengths with more than 10-GHz mode spacing because the laser pulse energy decreases as the repetition rate increases. At present, the reported widest mode spacing of a CEO-locked frequency comb with a mode-locked laser at telecommunications wavelengths is 750 MHz^41^. A recently reported CEO-locked EOM comb has achieved 10-GHz mode spacing using a continuous-wave (CW) laser stabilized with a high-finesse and low-expansion Fabry-Pérot cavity^42^. We experimentally demonstrated a simplified method for achieving a CEO-locked frequency comb with 25-GHz mode spacing, in which a free-running CW laser diode (LD) instead of an ultra-stable laser is used as a seed light source without stabilization to an external reference Fabry-Pérot cavity (see Fig. 1a). We measured the CEO beat signal by using a collinear 2*f* -to-3*f* SRI with a dual-pitch (DP) periodically poled lithium niobate (PPLN) ridge waveguide^43^. We observed a signal with about a 24-dB signal-to-noise ratio (SNR) at an RF spectrum analyzer set to a 100-kHz-resolution bandwidth. The full-width-at-half-maximum linewidth of the CEO signal is 1 MHz, which indicates that the CEO signal has large phase fluctuation. Therefore, by frequency-dividing the CEO signal, the phase fluctuation is decreased, and then the SNR can further increase. Figure 4a shows the CEO spectra measured with an RF spectral analyzer, which are frequency-divided by 1 (black), 8 (blue), and 16 (red), respectively. As a result of frequency-dividing the CEO signal by 16, the SNR of the CEO signal increased to more than 30 dB and the linewidth was reduced to less than 100 kHz. The frequency-divided CEO signal was locked at the 20-MHz reference frequency by controlling the center frequency of the LD with a feedback circuit and an external RF reference signal. We were able to achieve a CEO-locked EOM comb with 25-GHz mode spacing in the telecommunications wavelength region, which has been standardized in ITU-T. Figure 4b shows the CEO phase noise measured with a signal source analyzer with a low noise floor and cross-correlation measurement (E5052B + E5053A, Keysight Technology). Without the CEO locking, it was difficult to measure the phase noise at low offset frequency because of carrier frequency fluctuation. With the CEO locking, the phase noise at offset frequencies less than 3 kHz is suppressed. This means that the feedback loop bandwidth corresponds to around 3 kHz. Next, we compared the phase noise with the CEO locking between the EOM comb and a commercially available mode-locked Er-doped fibre laser. We found that the level of the phase noise with the EOM comb is identical to that with the mode-locked Er-doped fibre laser at offset frequencies less than 3 kHz. We also compared the Allan deviation of CEO signals with the CEO locking between them and found that the Allan deviation with the EOM comb is almost the same as that with the mode-locked Er-doped fibre laser (see Fig. 4c). The red circle and dotted line show the measured Allan deviation for the CEO frequency divided by 32 and the calibrated Allan deviation for the CEO frequency with the EOM comb. In this experimental setup, the SNR of the CEO signal is not sufficiently high, we measured the Allan deviation using the CEO signal frequency-divided by 32. If the SNR of CEO signal can be increased, the EOM comb could be stabilized without frequency division.

## Conclusion

We demonstrated an optical-referenceless AOF counter that achieves twelve-digit accuracy due to a record phase-noise reduction in widely used commercial microwave SGs. This unprecedented method can directly measure an AOF at twelve-digit accuracy with an RF frequency counter by just delivering a frequency-unknown laser into a phase modulator without relying on any optical reference source. Whenever we have a standard RF signal and an RF frequency counter, it is possible to measure an AOF with high accuracy. By magnifying the SG’s phase noise in the optical frequency region with an EOM comb and feeding it back to the SG, the SG’s phase noise can be greatly reduced. The near future will see the launch of a time delivery service that distributes time with higher accuracy by using an optical lattice clock as a master clock and an optical fibre network. Our method can easily and directly convert the time/phase-synchronization information engraved on the optical clock to a microwave frequency with higher accuracy, and the time/phase-synchronization technique with high accuracy will have an extremely large impact in the application fields of radar systems^1, 2^, wireless telecommunications, high-frequency transactions^5^, and electrical power systems such as smart grids^9^.

## Methods

### 25-GHz EOM comb generation

Our laser system generates a 25-GHz pulse train. We use two kinds of seed laser sources. One is a narrow linewidth laser (see Section “Introduction”) with a center wavelength of 1,542 nm and a linewidth of 1 Hz, which is stabilized with a reference cavity. The other is a free-running CW LD (see Section “AOF measurement without an optical reference”).The phase and intensity of the light from the narrow linewidth laser are modulated with eight conventional phase modulators driven by a sinusoidal-RF signal from an external RF synthesizer (YIG oscillator) at a modulation frequency of 25 GHz (see orange sine wave in Fig. 1a). The linear part of the down-chirping is then compressed to a short pulse train by propagating it in dispersive media. The applied modulation index obtained with the phase modulators is 32 π. The spectral bandwidth is around 39 nm. Next, it is necessary to increase the laser pulse energy. We reduce the ASE noise with a filter cavity^44^, which has a low-finesse (~ 1000) Fabry-Pérot cavity to allow light to transmit over a wide frequency bandwidth. And since the average output power of the EDFA is limited, we use an optical gate to increase the peak intensity of the amplified pulse. We then amplify the optical pulse at a 1.25-GHz repetition rate to 1 W. Finally, the chirped pulse can be compressed by the glass block in free space. We estimated the pulse width after the glass block to be 142 fs at 1.25 GHz.

### CEO stabilization for optical frequency counter

We generate more than 2/3-octave-wide SC spectrum by using a 40-cm-long highly nonlinear fibre with 0.8-nJ laser pulse energy. Therefore, the CEO signal can be measured by interfering second- and third-harmonic light at the wavelength of 600 nm. A 2*f*-to-3*f* SRI is useful for stabilizing a CEO frequency that has only a 2/3-octave bandwidth of the SC spectrum. For detecting the CEO signal in the 2*f*-to-3*f* SRI with high efficiency, we fabricated a DP-PPLN ridge waveguide^43^, which consists of two monolithically integrated segments with different quasi-phase matching (QPM) pitch sizes. Since we can reduce the Fresnel reflection and coupling losses by using this waveguide, we can detect the CEO signal with high efficiency. We observed it with about a 24-dB SNR at an RF spectrum analyzer set to a 100-kHz-resolution bandwidth. The linewidth of the CEO signal is 1 MHz. We found that this CEO signal has large phase fluctuation, because the SNR can be improved and the linewidth can be narrowed by dividing it by 32. On the other hand, SG 2 generates a 130-MHz reference signal. Both the CEO and SG 2 signals are divided by 32. The phase difference between the CEO and SG 2 signals is then measured with the phase detector inside SG 1. The voltage in the YIG-oscillator-based VCO inside SG 1 is adjusted to obtain a zero phase difference. Finally, the CEO is stabilized at 130 MHz, and the repetition rate becomes the frequency expressed as $\frac{{f}_{s}-{f}_{ceo}}{{N}^{\prime}}\times \frac{25 GHz}{1.25 GHz}$(see main text, Fig. 3).

## Supplementary Information

Supplementary Information.

## Data Availability

The datasets used and/or analysed during the current study are available from the corresponding author on reasonable request.
