# Supplementary material for: Optical-referenceless optical frequency counter with twelve-digit absolute accuracy
Source: Sci Rep. 2023 May 30;13:8750. doi: 10.1038/s41598-023-35674-8 (PMC10229652; doi:10.1038/s41598-023-35674-8)
Supplement: Supplementary file 1 — Supplementary Information. [file 41598_2023_35674_MOESM1_ESM.docx]

**Supplementary Information**

**Optical-referenceless optical frequency counter with twelve-digit absolute accuracy**

Atsushi Ishizawa*^,1, 3^, Tadashi Nishikawa^2^, Kenichi Hitachi^1^, Tomoya Akatsuka^1^, and Katsuya Oguri^1^

^1^*NTT Basic Research Laboratories, Nippon Telegraph and Telephone Corporation, 3-1 Morinosato Wakamiya, Atsugi, Kanagawa 243-0198, Japan*

^2^*Tokyo Denki University, Department of Electronic Engineering, 5 Senjyu-Asahi-cho, Adachi-ku, Tokyo 120-8551, Japan
^3^Currently at College of Industrial Technology, Nihon University, 1-2-1 Izumi-cho, Narashino, Chiba 275-8575, Japan*

*To whom correspondence should be addressed. E-mail: ishizawa.atsushi@nihon-u.ac.jp

**Block diagram of SG1**

Figure S1 shows a block diagram of SG 1. A YIG-oscillator-based VCO is used in SG 1. The YIG oscillator achieves high stability by using a permanent magnet. The drive current noise of the YIG oscillator with a permanent magnet can become much smaller than with an electromagnet. In our method, all SGs (SG 2 and SG 3) other than SG 1 are synchronized with the reference signal from the GPS-disciplined BVA OCXO (10 MHz). The YIG oscillator generates the microwave signal at 6.25 GHz. By frequency multiplying the microwave signal by four, a 25-GHz millimetre wave is generated. The phase noise *φ*_1_ (t) of the 25-GHz output signal on the YIG oscillator without PLL feedback is shown in Fig. S2. The 25-GHz microwave signal is delivered to the IM and PMs, and then the EOM comb with 25-GHz mode spacing is generated. By using a 2*f*-3*f* SRI, CEO signal can be measured. The CEO signal is connected to the CEO SIGNAL INPUT in Fig. S1.
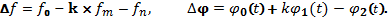
SG 2, which is synchronized with the GPS signal, provides the RF reference signal. Figure S3 shows the phase noise of SG 2 synchronized with the GPS reference signal, which is much lower than the phase noise of the CEO signal. Since the CEO signal has large phase fluctuation, which exceeds one cycle of the reference signal. The phase detector cannot compare the phase difference between the CEO SIGNAL INPUT and the 130-MHz (or 120-MHz) signal of the SG 2. Therefore, by dividing the CEO signal by 32, the phase detector determines the relative phase difference between the CEO SIGNAL INPUT and the 130-MHz (or 120-MHz) signal of the SG 2. The YIG-oscillator-based VCO inside SG 1 is adjusted so that the phase difference becomes zero. By the setting of the loop filter, the frequency variable range of SG 1 is limited within ± 2.4 MHz so as not to jump mode number $N$ at fixed CEO frequency. Therefore, the comb mode number always stays the same value during the feedback control of the VCO in our experiment.


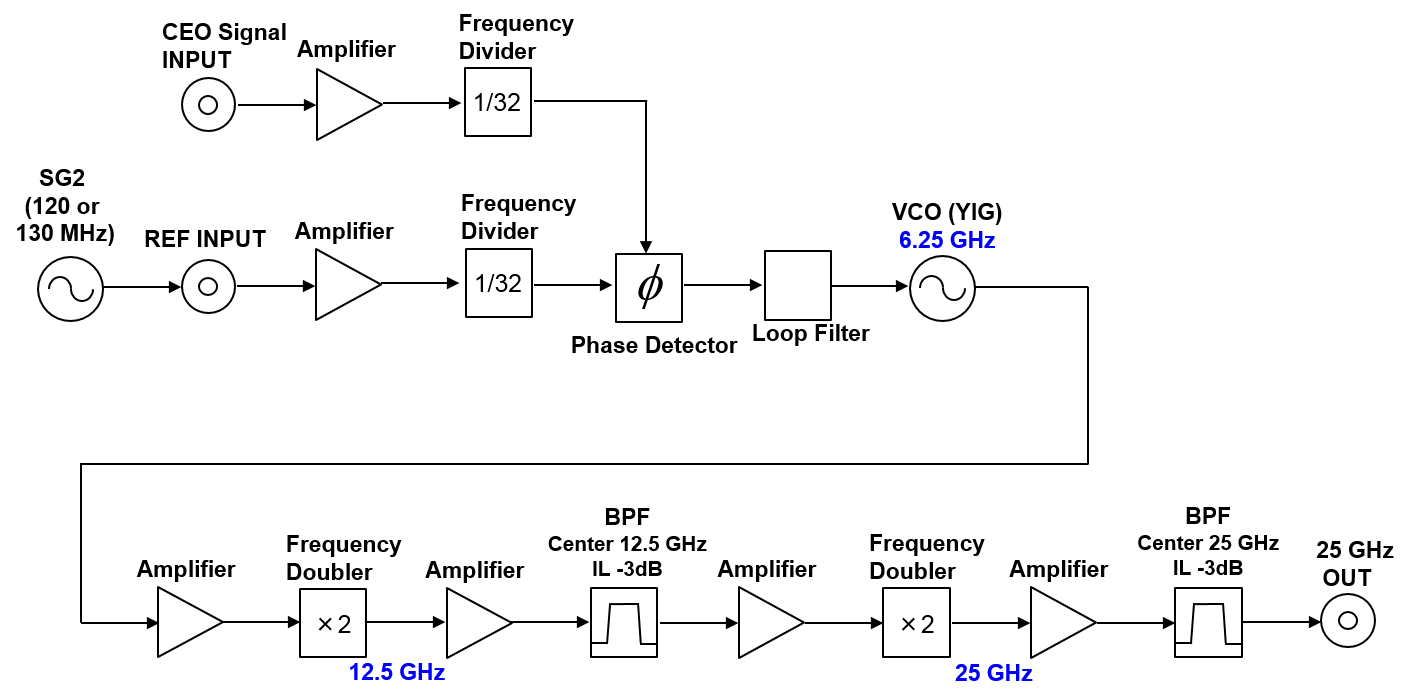


**Figure S1. Block diagram of SG 1.**

VCO (YIG) is the YIG-oscillator-based VCO, and it generates the low-phase-noise millimetre wave at 25 GHz. BPF: Bandpass filter. IL: Insertion loss.

**Figure S2. Phase noise of the YIG-oscillator-based VCO at 25 GHz without PLL feedback.** The phase noise at offset frequencies from 10 Hz to 1 MHz is shown. The average number is 16.

**Figure S3. Phase noise of SG 2 at 130 MHz, which is synchronized with the the reference signal from the GPS-disciplined BVA OCXO.** The phase noise at offset frequencies from 10 Hz to 1 MHz is shown. The average number is 16.
